# Potential of Aromatic Plant-Derived Essential Oils for the Control of Foodborne Bacteria and Antibiotic Resistance in Animal Production: A Review

**DOI:** 10.3390/antibiotics11111673

**Published:** 2022-11-21

**Authors:** Lianhua Zhang, Fei Gao, Junwei Ge, Hui Li, Fei Xia, Hongtong Bai, Xiangshu Piao, Lei Shi

**Affiliations:** 1 Key Laboratory of Plant Resources, Institute of Botany, Chinese Academy of Sciences, Beijing 100093, China; 2 China National Botanical Garden, Beijing 100093, China; 3 University of Chinese Academy of Sciences, Beijing 100049, China; 4 College of Pharmacy, Shandong University of Traditional Chinese Medicine, Jinan 250355, China; 5 State Key Laboratory of Animal Nutrition, College of Animal Science and Technology, China Agricultural University, Beijing 100193, China

**Keywords:** essential oils, antibacterial property, foodborne bacteria, antibiotic resistance, animal production

## Abstract

Antibiotic resistance has become a severe public threat to human health worldwide. Supplementing antibiotic growth promoters (AGPs) at subtherapeutic levels has been a commonly applied method to improve the production performance of livestock and poultry, but the misuse of antibiotics in animal production plays a major role in the antibiotic resistance crisis and foodborne disease outbreaks. The addition of AGPs to improve production performance in livestock and poultry has been prohibited in some countries, including Europe, the United States and China. Moreover, cross-resistance could result in the development of multidrug resistant bacteria and limit therapeutic options for human and animal health. Therefore, finding alternatives to antibiotics to maintain the efficiency of livestock production and reduce the risk of foodborne disease outbreaks is beneficial to human health and the sustainable development of animal husbandry. Essential oils (EOs) and their individual compounds derived from aromatic plants are becoming increasingly popular as potential antibiotic alternatives for animal production based on their antibacterial properties. This paper reviews recent studies in the application of EOs in animal production for the control of foodborne pathogens, summarizes their molecular modes of action to increase the susceptibility of antibiotic-resistant bacteria, and provides a promising role for the application of nanoencapsulated EOs in animal production to control bacteria and overcome antibiotic resistance.

## 1. Introduction

Today, the world produces more than three times the quantity of meat as it did 50 years ago. In 2018, meat production was around 340 million tons [1]. By 2050, a 102% increase in the food supply will be necessary to meet the demand [2]. However, in livestock and poultry production, animals usually face various stressors, such as oxidative processes, nutritional imbalances, allergens, pathogenic bacteria, etc., which could result in diarrhea, growth retardation, and high morbidity and mortality. The conventional way to maintain or improve yield in animal products was to use antibiotic growth promoters (AGPs) at subtherapeutic levels. Unfortunately, exposure to AGPs in the early stages of life adversely affects the development of the immune function and intestinal bacteria and ultimately results in increased susceptibility to infections and diseases in livestock [3]. Antibiotic abuse in food-producing animals is a primary reason for the selectivity and diffusion of antibiotic resistance and disease outbreaks induced by resistant foodborne bacteria, which could create foodborne risks to human health [4]. Although the addition of AGPs to diets has been forbidden in some countries to promote the growth performance of food-producing animals, antibiotic resistance is still widespread in the world [5]. As animal production is one of the main sources of antibiotic resistance genes due to the selective pressure that occurs in this environment, the presence of mobile genetic elements in the intestinal bacteria could spread antibiotic resistance [6]. Animal-derived foods contaminated with resistant bacteria can lead to serious infections and diseases that are difficult to treat. Globally, an estimated 2 million people receive treatment for resistant infections each year, with approximately 700,000 deaths and treatment costs exceeding 100,000 million dollars [1]. Such data demonstrate the economic and social consequences of the emergence of resistant foodborne pathogens–an aftereffect of the misuse of antibiotics in animal production. It is necessary to seek novel feed additives for maintaining the efficiency of livestock production and reducing the spread of drug-resistant pathogens, which is beneficial to human health and the sustainable development of animal husbandry.

Essential oils (EOs) are becoming potential antibiotic alternatives due to their natural origin, low toxicity, and free of residues [7]. EOs are a mixture of various volatile compounds extracted from aromatic plants (flowers, fruits, seeds, stems, leaves, etc.) [8]. Lamiaceae are one of the most important families of plant EOs with antibacterial effects, among which oregano, thyme, and rosemary have been widely used in the food industry [9,10,11]. Several in vivo studies indicated that EOs increased *Lactobacillus* abundance and decreased *Escherichia coli* or total coliforms in piglets [12,13,14]. These results were similar to those of several studies of poultry supplemented with EOs [15,16,17], suggesting that EOs resulted in some fundamental changes within the gut microbiota, primarily the observed numbers of *Lactobacillus* species. In addition to the antibacterial property, EOs also exhibit other biological activities, including anti-inflammatory, antioxidant, anti-tumor, and immune-regulating properties [1], suggesting that EOs could improve production performance in animals. Franz et al. [18] and Windisch et al. [19] reviewed that the average improvement in weight gain, feed intake, and feed conversion caused by EOs was 2.0, 0.9, and 3.0% for piglets and 0.5, 1.6, and −2.6% for poultry, respectively. However, the use of aromatic plant-derived EOs in grower–finisher pigs appears unsuccessful. Janz et al. [20] and Yan et al. [21] did not observe any improvement in the growth performance induced by EOs in finisher pigs. The different results may be caused by different digestive physiology, sources of aromatic plant EOs, the quantity used in the diet, and the environmental conditions used in the experiments.

The indiscriminate use of antibiotics in animal production plays an important role in the antibiotic resistance crisis and foodborne disease outbreaks. Since antibiotic resistance remains a major threat to public health, most research has focused on the causes, threats, and management strategies related to human health, leaving aside aspects of animal production. This paper collects the research progress of the use of EOs in animal production to control foodborne pathogens, summarizes their molecular modes of action with respect to antibiotic resistance, and provides a promising role for the application of nanoencapsulated EOs in animal production to more effectively control bacterial infections and overcome antibiotic resistance.

## 2. Foodborne Pathogenic Bacteria and Antibiotic Resistance

Undoubtedly, the discovery of AGPs is one of the greatest inventions of the 20th century, as they could improve growth performance and combat infectious diseases in livestock. Globally, the total consumption of antimicrobials in animal production, including pigs, chickens, and cattle was 93,309 tons in 2017 and is expected to grow by 11.5% to 104,079 tons by 2030 [22]. With the excessive use and abuse of AGPs or antibiotics used to treat infections in humans and food-producing animals worldwide, there is a greater chance for bacteria to develop complicated resistances against antibiotics [23]. Antibiotic resistance is defined as a natural process of selecting resistant microorganisms that could thrive in the environment and multiply and perpetuate resistance characteristics [24]. Moreover, antibiotic resistance could spread from food-producing animals to humans by eating meat, milk, and their products [25,26]. Therefore, animal-derived foods contaminated with drug-resistant pathogens could result in severe infections and untreatable diseases in humans. If no action is taken, antibiotic resistance is expected to cause more deaths than cancer by 2050, which could be a massive threat to human health [27]. Increased emergence of *E. coli* infection has been shown to result in higher morbidity and mortality in weaned pigs [28]. According to an estimate, 80 species of bacteria, including *E. coli* and *Salmonella*, could pose a severe threat to poultry production [29]. Mastitis, induced by *Staphylococcus aureus*, *Streptococcus agalactiae*, *Corynebacterium bovis*, *Streptococcus uberis*, *Streptococcus dysgalactiae*, *E. coli*, *Serratia marcescens* and *Proteus mirabilis* in dairy cows, has been an economic welfare problem for dairy farms [30,31,32]. To date, many studies have reported main antibiotic resistant bacteria from different animal-derived foods in some countries, including *E. coli* (Table 1), *Salmonella* spp. (Table 2), *Staphylococcus* spp. (Table 3), and *Listeria* spp. (Table 4). These data denote that foodborne antibiotic resistance is a widespread problem worldwide-a result of the abuse of AGPs in animals. To ensure human health and animal food safety, new and multidimensional approaches are needed to control bacterial infections for animal production. Here, we discuss the trend and development of EOs and their individual compounds as alternatives to antibiotics to address antibiotic resistance.

## 3. EOs and Their Individual Compounds Derived from Common Aromatic Plants and Their Antibacterial Actions

**Figure 1:**
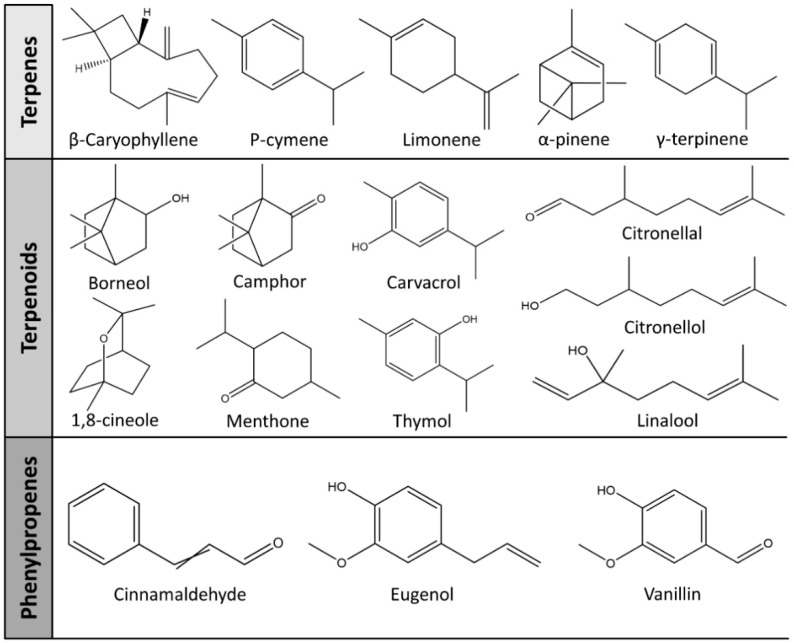
Chemical structures of major components in aromatic plant-derived EOs.

**Figure 2:**
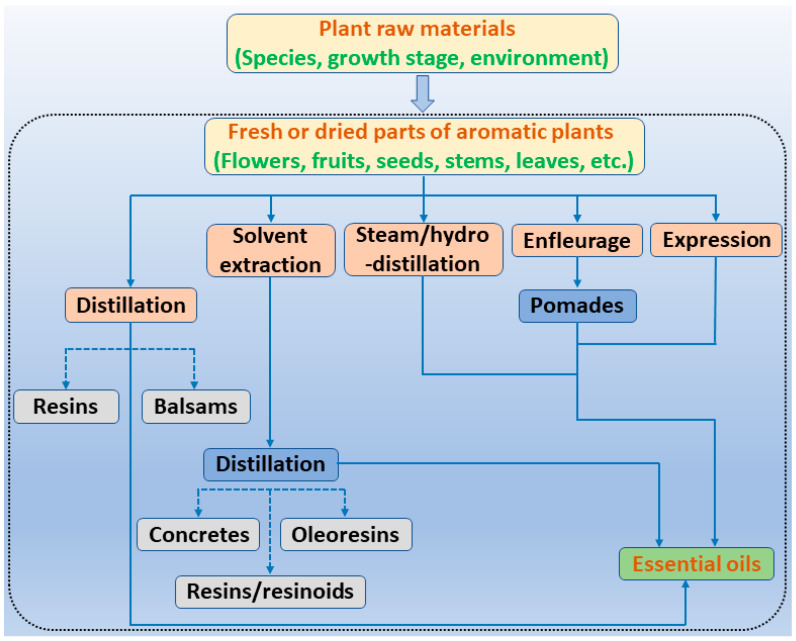
The extraction process of aromatic plant-derived EOs (adapted from [72]).

It has been reported that there are 3000 species of aromatic plants widely distributed in European countries along the Mediterranean coast, as well as in China, India, Central Asia, and South America [71], which are mainly concentrated in Apiaceae, Asteraceae, Lamiaceae, Lauraceae, Myrtaceae, Poaceae, Rutaceae, and Zingiberaceae. The most well-known species are from the genera *Origanum*, *Rosmarinus*, *Thymus*, and *Ocimum*, and all belong to the family Lamiaceae. These species are most commonly used to produce EOs due to the high content of aromatic compounds, where variable chemical compositions are divided into mainly three categories, namely terpenes, terpenoids, and phenylpropenes (Figure 1). The changes in various active ingredients and their content in EOs are mainly related to the raw materials of the plants and the extraction process (Figure 2). In general, two or three active ingredients in EOs have relatively high proportions, ranging from 20% to 70%, which contribute to the primary property of the mixture [8]. The utilization of EOs and their individual compounds in livestock production is considered a promising alternative to antibiotics for bacterial control. In the antibacterial evaluation system, the minimum inhibitory concentration (MIC) is an essential indicator for evaluating the antibacterial properties of EOs and their individual compounds. Briefly, EOs were dissolved at two-fold serial dilutions and the MIC was considered as the lowest concentration of EOs at which no visible bacterial growth was observed. Numerous in vitro studies have reported the antimicrobial properties of EOs and their individual compounds against common pathogens in animal product processing environments, including *E. coli*, *Salmonella* spp., *Staphylococcus* spp., and *Listeria* spp. Table 5 and Table 6 summarize the antimicrobial activity of EOs and their active components from aromatic plants according to the MIC values.

The unique antimicrobial effects of plant EOs depend on their active components and are associated with the functional groups and structural arrangement of their active molecules, while different chemical components often have a synergistic antibacterial effect. Among the main components of EOs, phenols and aldehydes have the most potent antimicrobial activity, followed by alcohols, ketones, esters, and hydrocarbons [104]. Previous studies have shown that lipophilicity and the existence of phenolic hydroxyl, methoxy, and olefin bonds play a vital role in the antibacterial ability of active compounds in EOs, because these functional groups could consume proton motive force, affect intracellular pH value, and disrupt the oxidative phosphorylation of bacteria [105]. Many individual components of EOs with the essential functional groups mentioned above, including carvacrol, thymol, cinnamaldehyde, and eugenol have significant bactericidal activity. Therefore, EOs derived from aromatic plants, including oregano, thyme, cinnamon, and clove have potent antibacterial activity due to their high content of these compounds.

EOs have different actions against bacteria. Most EOs target bacterial cell walls, which also explains that EOs have a better ability to suppress gram-positive bacteria compared to gram-negative bacteria [106,107]. EOs and their components also change the fatty acid profile of the cell membrane, damage the cytoplasmic membrane, consume the proton motive force, reduce the synthesis of adenosine triphosphate (ATP) and increase ATP hydrolysis, and decrease membrane potential [108,109,110,111,112,113]. The hydrophobicity of EOs could increase membrane permeability, which further results in the leakage of bacterial cell content, including potassium ions and genetic materials [114,115]. For example, *Origanum compactum* EO (mainly carvacrol, thymol, and *p*-cymene) could alter the integrity of the cell membrane and increase the permeability of the membrane, leading to the leakage of genetic materials in *Bacillus subtilis* [114]. In some instances, EOs could also alter membrane permeability by disrupting electron transport systems [116]. Some EOs, especially those rich in phenolic compounds, can enter the phospholipid bilayer of cell membranes and interact with membrane proteins to disrupt the normal physiological activities of bacteria [108]. Alterations in membrane permeability and the disruption of molecular and ion transportation leads to imbalances within bacterial cells, which could induce the denaturation of cellular enzymes and proteins, leakage of ions and metabolites, and the solidification of the cytoplasm [117]. ATP is important for bacterial respiration and metabolism, and could be influenced by EOs and their components. For example, cinnamon oil and its active compound, cinnamaldehyde, decrease intracellular ATP levels in *Mycobacterium avium* subsp. *paratuberculosis* [118]. Quorum sensing (QS) is an intercellular communication system that allows bacteria to secrete and detect external signaling molecules, which could promote the development of virulence factors and biofilms and the production of secondary metabolites. Gram-positive bacteria use auto-inducing peptides (AIPs) for signaling, whereas gram-negative bacteria use *N*-acyl-homoserine lactones (AHLs). Terpenes and phenylpropenes such as carvacrol, thymol, eugenol, and cinnamaldehyde have anti-biofilm and anti-QS properties against bacteria [119]. Carvacrol and thymol have been shown to inhibit new and existing biofilms of pathogenic bacteria, such as *L. monocytogenes* and *Pseudomonas aeruginosa* [120,121]. Cinnamaldehyde could suppress biofilm formation in pathogenic bacteria, including *Staphylococcus epidermidis*, *L. monocytogenes*, and *Cronobacter sakazakii* [121,122,123]. Eugenol could suppress the production of QS-regulated violacein in *Chromobacterium violaceum* and virulence factors in *P. aeruginosa* [124]. Several aromatic plant-derived EO components have specific anti-QS strategies through binding to LuxR-type AHL receptor proteins and LuxI-type AHL synthases, which are present in terpenes and phenylpropenes, including carvacrol, thymol, cinnamaldehyde, and eugenol [125]. Collectively, the antibacterial activity of EOs may not rely on a single mechanism due to the complexity of the active components, so pathogenic bacteria are less likely to develop resistance to EOs.

Given the above antibacterial mechanisms, EOs have the potential to replace AGPs in animal production. Interestingly, several studies have shown that EOs and their components have synergistic effects on controlling resistant bacteria when combined with antibiotics. *Origanum vulgare* EO (mainly carvacrol, β-caryophyllene, and γ-terpinene) has synergistic effects against multidrug-resistant *Acinetobacter baumannii* when combined with polymyxin B [126]. *Thymus zygis* EO (mainly thymol, carvacrol, and *p*-cymene) has synergistic effects with ciprofloxacin, ampicillin, or vancomycin against *S. aureus*, and could change the phenotype from antibiotic resistance to antibiotic susceptibility [127]. Carvacrol, thymol, eugenol, and α-pinene have shown synergistic interactions with tetracyclines and gentamicin against pathogenic bacteria, including *E. coli*, methicillin-resistant *S. aureus*, and *P. aeruginosa* [128]. Moreover, carvacrol has synergistic effects on suppressing erythromycin-resistant Group A Streptococci when combined with erythromycin [129]. Cinnamaldehyde synergistically increases the antibiotic susceptibility of *E. coli* to tetracyclines and erythromycin [130]. Therefore, in addition to being used as antibiotic alternatives to boost animal production, EOs could prevent the development of bacterial resistance, which has important implications for animal production and human health. In the following content, this review summarizes antibiotic resistance genes (ARGs) and the effects of EOs and their components on antibiotic resistance.

## 4. Antibiotic Resistance Genes and the Impact of EOs and Their Individual Compounds on Antibiotic Resistance

Food-producing animals are a major source of ARGs. ARGs can be transferred to humans primarily through the ingestion of animal-derived foods [131]. Many genes associated with bacterial resistance are present in chromosomes and certain plasmids [132]. Numerous studies reported ARGs of foodborne pathogens, such as *E. coli*, *Salmonella* spp., *Staphylococcus* spp., and *Listeria* spp. ARGs and their resistance to the AGPs of *E. coli*, *Salmonella* spp., *Staphylococcus* spp., and *Listeria* spp. are shown in Table 7 [31,35,38,60,133,134,135,136,137,138,139,140,141,142,143,144]. According to the data, different species have their own ARGs. Notably, shared genes between different species may result from interspecific communication of ARGs among bacteria [145].

As mentioned earlier, EOs and their components have synergistic effects against resistant bacteria when combined with antibiotics, indicating that EOs and their individual compounds may increase antibiotic susceptibility of drug-resistant bacteria. The determinant of multidrug resistance is through the increased expression of the efflux pump genes, which could lead to a decrease in the antibiotic concentration of bacteria and an increase in the MIC values of antibiotics [146]. The efflux pump is a transport protein associated with intercellular communication and biofilm formation, which could protect bacteria by pumping large amounts of AGPs out of cells [147]. Recently, several studies have reported that EOs and their individual compounds could suppress the activities of bacterial efflux pumps [148]. For example, *Origanum vulgare* EO (pulegone, 1,8-cineole, and borneol) and *Thymus daenensis* EO (carvacrol, γ-terpinene, and α-terpinene) could inhibit the activity of the PmrA efflux pump at sub-MIC levels against fluoroquinolone-resistant *Streptococcus pneumoniae* [149]. *Satureja khuzistanica* EO (mainly thymol and carvacrol) has been shown to reduce the expression of *mexY* and *mexE* efflux pump genes in *P. aeruginosa* [150]. *Rosmarinus officinalis* EO (mainly α-pinene, γ-sitosterol, and 1,8-cineole) could decrease the expression of the *bla_TEM_* efflux pump gene in *A. baumannii* and *P. aeruginosa*, and the *bla_OXA-23_* efflux pump gene in *A. baumannii* [151]. *Chenopodium ambrosioides* EO (mainly α-terpinene, *p*-cymene, and ascaridole) and its main constituent, α-terpinene could suppress the activity of the TetK and NorA efflux pumps in *S. aureus* [152]. Similarly, *Salvia fruticosa* EO could markedly inhibit the activity of the TetK efflux pump in tetracyclines-resistant *S. epidermidis* [153]. *Piper caldense* EO (mainly caryophyllene oxide, δ-cadinene, and spathulenol) has the potential to suppress the MepA, NorA, and QacC efflux pumps of multidrug-resistant *S. aureus* [154]. The EtBr efflux inhibition analysis has been used to evaluate the inhibitory activity of EOs and their components in efflux pumps. The EtBr efflux inhibition assay showed that *Cuminum cyminum* EO (mainly cuminic aldehyde, γ-terpinene, α,β-dihydroxyethylbenzene, 2-caren-10-al, and β-pinene) could significantly inhibit the activity of the NorA efflux pump in *S. aureus* [155]. For the main active components of EOs, carvacrol, thymol, and eugenol could inhibit EtBr efflux through active pumps from *E. coli*, *S.* Typhimurium, *S.* Enteritidis, and *S. aureus* [156].

In addition to inhibiting the activities of efflux pumps in bacteria, some EOs and their components could inhibit gene expressions related to virulence factors and have anti-plasmid conjugation potential for bacteria. *Cinnamomum camphora* EO (mainly linalool, cineole, and sabenene) could inhibit the expressions of QS-regulated virulence genes such as *lasA*, *lasB*, *pilE3*, *vioA*, *vioB*, *vioC*, *vioD*, *vioE*, and *hmsHNFR* in *Chromobacterium violaceum* [157]. Lemongrass EO (mainly geranial, neral, limonene, and geraniol) could down-regulate the expression of genes related to virulence factors such as *hly*, *inlB*, *inlC*, *inlJ*, *plcA*, *plcB*, and *lmo2470* in *L. monocytogenes* [158]. Carvacrol, an important component of *Origanum vulgare* EO, has been shown to down-regulate the expression of genes related to virulence factors such as *ctxB*, *hlyA*, *tcpA*, and *toxT* in *Vibrio cholerae* [159]. Eugenol could decrease the content of virulence factors, including rhamnolipid and pyocyanin, and inhibit related gene expression such as *rhlA* in *P. aeruginosa* [160]. Moreover, the single constituents of *Thymus vulgaris* EO, including thymol, linalool, *R*-carvone, eugenol, eucalyptol, *S*-carvone, and borneol, have anti-plasmid conjugation potential such as decreasing the transfer of plasmid pKM101, which could reduce virulence and spread of resistance in *E. coli* [161].

Given that animal-derived foods are one of the main sources of ARGs, some EOs and their components could not only replace antibiotics to improve animal performance and gut health, but may also have great potential to alleviate the widespread problem of antibiotic resistance in animal production. However, much research is needed in the future to study and elucidate this strategy.

## 5. Nanoencapsulated EOs as a Promising Option for Animal Production against Antibiotic Resistance

Most bacteria grow as single planktonic cells or communities within the biofilm. As the main form of bacterial survival, the biofilm is a bacterial community encased in a self-generated extracellular polymer matrix that provides the community with a variety of competitive advantages, including increased resistance to various stress stimuli [162]. Moreover, bacterial biofilms facilitate horizontal gene transfer through the exchange of genome fragments and mobile genetic elements in bacteria, which could contribute to the spread of ARGs [163]. The extreme tolerance of bacterial biofilms to antibiotics is particularly problematic because it makes it more challenging to fight antibiotic-resistant bacteria. Mixed bacterial biofilms have been observed in intestinal diseases, most of which are pathogenic. Biofilm-related pathogens have become a severe problem not only in humans but also in animal production. In host-microbiota interactions, bowel biofilms play a critical role in the pathogenesis of inflammatory bowel disease (IBD) and other infectious diseases in humans [164]. An invasive biofilm, which harbors the opportunistic pathogen *Bacteroides fragilis* as a crucial species, has been shown to be detected in patients with IBD [165]. Biofilm formations have also been observed in common antibiotic-resistant foodborne bacteria such as *E. coli*, *S. aureus, S.* Typhimurium, and *L. monocytogenes* [166,167]. The SslE protein, secreted by *E. coli*, degrades intestinal mucins, including MUC2, MUC3, and MUC5AC and accelerates biofilm maturation, which is a significant factor in the infection process of highly virulent species [167]. Enterotoxigenic *E. coli*, a virulent strain, causes severe infections and diarrheal diseases in animals, including weaned pigs, and is closely related to increased mortality and severe impairments in production [20]. *S.* Typhimurium could decrease the expressions of Occludin and Claudin-1 and subsequently disrupt the intestinal epithelial barrier in broiler chickens [168]. Peptidoglycan from *S. aureus* induces intestinal inflammation and disrupts intestinal barrier functions through TLR2-regulated activation of the NF-κB pathway in porcine jejunal epithelial cells [169]. During foodborne infection, *L. monocytogenes* cross the intestinal mucosal barrier via *Listeria* adhesion protein, which could break down the epithelial tight junction barrier for bacteria to enter the lamina propria [170]. The loss of a critical protective barrier facilitates the migration of pathogenic bacteria across the epithelial barrier and biofilm information [171]. Furthermore, biofilms provide a favorable environment for pathogens to escape host defenses, further promoting the occurrence and progression of intestinal diseases [172]. Collectively, bowel biofilms dominated by antibiotic-resistant foodborne bacteria are of great significance in the development of intestinal disorders and the transition to the pathogenic microbiota, which could further induce food safety problems of animal origin and harm public health.

Concerns about the potential for antibiotic resistance to transfer from animal intestinal bacteria to humans through the consumption of animal-derived foods calls for alternatives to antibiotics in animal production. This review focuses on the advance of novel antibacterial agents, particularly those effective against the strong resistance of bacterial biofilms. EOs and their individual compounds are effective against resistant bacterial infections and are expected to decrease the selection and spread of AGRs [173]. As the main form of bacterial survival, bacterial biofilm information could increase antibiotic resistance by 10–1000 than their planktonic counterpart, which is the main cause of bacterial resistance [174,175]. However, the lipophilic properties of EOs and their individual compounds generally severely limit their applications due to their low permeability and poor absorption under aqueous biological and non-biological conditions such as biofilm matrices [176]. Therefore, the regular applications of EOs and their individual compounds may be ineffective against resistant bacterial infections because of their poor permeability to the biofilm matrix and extracellular polymer materials. The limitation could be overcome by developing nanoencapsulation methods, including polymeric nanocapsules, nanostructured lipid particles, liposomes and nanoemulsions, and other nanosystems [176]. Biomolecule-based nanoencapsulation can be designed and engineered for antimicrobial agents to surmount current and classical challenges, including the emergence of multidrug-resistant bacteria, the inefficiency and applicability limitations of existing antimicrobial agents, and biofilm formation [177]. Nanoencapsulated EOs and their individual components could make the whole formation water-soluble, allowing nanosystems to easily penetrate water-filled channels and the cavities of bacterial biofilms [178]. In addition, nanodelivery systems could sustain and control EO release at the site of action and mask unpleasant tastes or odors of EOs to minimize unacceptable organoleptic effects [178,179]. Numerous studies have reported different nanocarrier systems with encapsulated common EOs and their individual components, including oregano oil, cinnamon oil, thyme oil, carvacrol, cinnamaldehyde, thymol, and eugenol (Table 8). These in vitro results summarize that nanodelivery systems containing EOs and their components as novel antibacterial agents could suppress biofilm formation and combat bacteria within mature biofilms on biotic and abiotic surfaces, indicating that nanoencapsulated EOs may be a prospective approach for controlling resistant bacterial biofilm-related infections and overcoming existing antibiotic resistance in animal production.

In animal-derived foods, chitosan nanoparticles containing mandarin EO could inhibit biofilm formation and destroy mature biofilms of *E. coli* and *S. aureus*, as well as have great potential for pork preservation [185]. Moreover, nanoencapsulated EOs used directly in animal food have been shown to be effective in reducing the rate of foodborne bacterial infections in animals. For in vivo studies, thymol nanoemulsion has been shown to upregulate the gene expression of *IgA*, *MUC2*, *IL-10*, and *FABP2*, and downregulate the gene expression of the vital virulence gene *invA* in a broiler chicken after a *S.* Typhimurium infection [191]. Chitosan nanoencapsulated thyme and cinnamon EOs could more effectively improve breast percentage, increase serum IgM and IgY contents, and improve intestinal *Lactobacillus* spp. abundances in broiler chickens compared with free EOs [17], with chitosan having its own benefits on growth rate due to its antioxidant and antibacterial properties and increased ileal digestibility of dry matter [192]. Chitosan nanoencapsulated garlic EO enhanced more evaluated parameters, including body weight gain, feed conversion ratio, intestinal *MUC2* gene expression, and the *Lactobacilli* population in broilers compared with free garlic EO [193]. Thyme EO loaded in chitosan nanoparticles could more effectively improve the feed conversion ratio and decrease the number of coliform and total aerobic bacteria in broilers compared to unencapsulated thyme EO [194]. Cumin EO in chitosan nanoparticles could more effectively improve growth performance, *MUC2* gene expression and sustain broiler immune responses compared with free-form cumin EO [195]. Together, EOs and plant extracts are mainly encapsulated bioactive substances and phytochemicals used in animal diets, and chitosan was found to be the most effective nanocarrier to load EOs and plant extracts [196]. Nanoparticles and nanocapsules are frequently studied nanocarriers, most of which are processed by the ionotropic/ionic gelation. However, nanofibers, nanohydrogels, and nanoemulsions have not been found yet for their application in feed bioactive substances. These nanocarriers have improved protection, stability, and controlled release of feed bioactive substances, which provides additional nutrition for the growth performance of livestock regardless of the low stability and water solubility of bioactive substances. However, like other emerging technologies, nanocarriers may threaten the health of animals and, ultimately, human consumers. The physicochemical properties of nanocarriers allow them to penetrate the physical barriers of enterocytes and put the animal at risk of gastrointestinal disease. At the same time, we lack a fundamental understanding of the behavior of nanocarriers in the biological system in terms of in vivo distribution at the cellular and organ levels. In addition, one of the biggest obstacles to commercializing nanoencapsulation technology in animal feed is legislation. Despite promising, more quantitative and in vivo studies should be performed before the commercial application of nanoencapsulated EOs as antibiotic alternatives.

## 6. Conclusions

**Figure 3:**
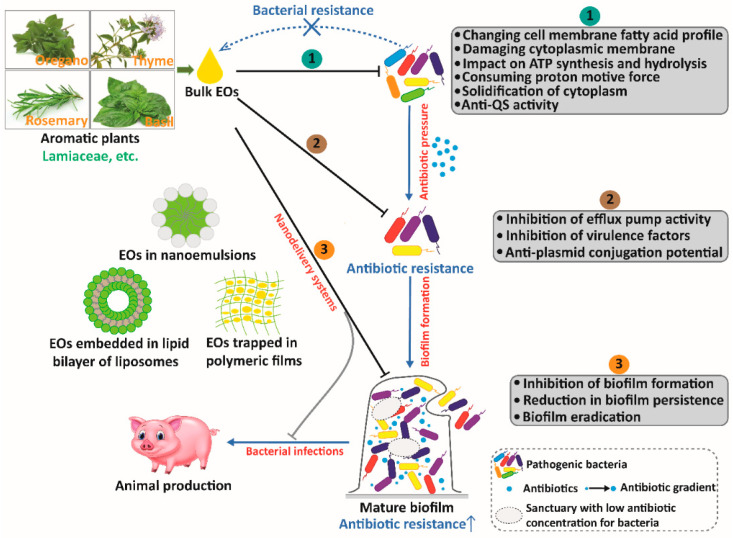
Summary of the potential of aromatic plant-derived EOs as antibacterial agents in animal production against antibiotic resistance.

Although dietary supplementation with AGPs at sub-therapeutic levels is an effective way to improve performance and prevent bacterial infections in animals, the abuse of AGPs could induce public risks, environmental contamination, and the diffusion of antibiotic-resistant bacteria. The prohibition of AGPs in feed is associated with many challenges in animal production, such as poor growth performance and severe intestinal diseases. Due to their powerful antibacterial properties, EOs and their individual compounds have emerged as novel antibiotic alternatives to combat bacterial infections. The successful application of EOs and their individual compounds is based on whether our understanding of how EOs work is based on sufficient research. As shown in Figure 3, this article reviews foodborne pathogenic bacteria and antibiotic resistance, and the impacts of EOs and their individual compounds on foodborne pathogenic bacteria and ARGs. In addition, nanotechnology provides a promising tool for the delivery of EOs and their individual compounds to the gut and for enhancing the effectiveness of EOs and their components in animal production. It should be noted that evidence for a link between EOs and antibiotic-resistant foodborne bacteria in animal production is incomplete, as in vitro studies could not directly demonstrate the impact of EOs and their individual compounds on alleviating antibiotic resistance in livestock production. Therefore, it is necessary to establish and strengthen extensive cooperation between academic research and the livestock industry, to meet the needs of experimental research, and to clarify the precise application mode and benefits of EOs and their individual compounds in animal production in a timely manner.

## Figures and Tables

**Table 1:** *Escherichia coli* isolated from animal-derived foods and their resistance to antibiotics.

Country	Sources	Antibiotic Resistance	References
Australia	Dairy cows	Amoxicillin-clavulanate, ceftiofur, cefoxitin, gentamicin	[33]
Brazil	Chicken carcass	Aminoglycosides, colistin, β-lactams, macrolides, quinolones, sulfonamides, tetracyclines, trimethoprim	[34]
Cambodia	Broiler carcass and pig carcass	Ampicillin, cefotaxime, ceftazidime, chloramphenicol, ciprofloxacin, streptomycin, tetracyclines, trimethoprim	[35]
China	Retail chicken and pork	Ampicillin/sulbactam, aztreonam, cefepime, cefotaxime, ciprofloxacin, colistin, doxycycline, gentamicin, levofloxacin, minocycline, piperacillin/tazobactam constant 4, polymyxin B, tigecycline, trimethoprim/sulfamethoxazole	[36]
Denmark	Pig carcass	Ampicillin, chloramphenicol, gentamicin, streptomycin, trimethoprim	[37]
Egypt	Raw dromedary camel milk	cefoxitin, erythromycin, novobiocin, piperacillin, rifampicin, rifamycin, streptomycin	[38]
Ethiopia	Chicken, goat, and beef meat	Ampicillin, chloramphenicol, erythromycin, gentamycin, streptomycin, tetracyclines, trimethoprim/sulfamethoxazole	[39]
Germany	Retail chicken meat	Ampicillin, cephalosporin, ciprofloxacin, nalidixic acid, streptomycin, tetracyclines, trimethoprim	[40]
Italy	Pig carcass	Ampicillin, chloramphenicol, gentamicin, streptomycin, tetracyclines, trimethoprim	[37]
South Africa	Raw meat	Ampicillin, ceftazidime, streptomycin, sulphafurazole, tetracyclines	[41]
Thailand	Broiler carcass and pig carcass	Ampicillin, cefpodoxime, ceftazidime, ciprofloxacin, gentamicin, sulfamethoxazole, tetracyclines, trimethoprim	[35]
United States	Dairy cattle	Azithromycin, ciprofloxacin, gamithromycin, tulathromycin	[42]
Vietnam	Retail raw foods (chicken, pork, fish, and shrimp)	Chloramphenicol, ciprofloxacin, gentamicin, nalidixic acid, streptomycin, tetracyclines, trimethoprim/sulfamethoxazole	[43]

**Table 2:** *Salmonella* spp. isolated from animal-derived foods and their resistance to antibiotics.

Country	Sources	Antibiotic Resistance	References
Brazil	Fresh tilapia fillets	amoxicillin/clavulanic acid, chloramphenicol, sulfonamide, tetracyclines	[44]
Cambodia	Retail poultry	Amoxicillin, cefalotin, chloramphenicol, cotrimoxazole, nalidixic acid, streptomycin, sulfonamide, tetracyclines, ticarcillin	[45]
China Egypt Iran	Pork, chicken, duck, and fish	Ampicillin, streptomycin, tetracyclines	[46]
	Chicken meat	Amoxicillin, ampicillin, erythromycin, nalidixic acid, oxytetracyclines, penicillin, sulfamethoxazole	[47]
	Chicken meat	Difloxacin, erythromycin, florfenicol, flumequine, lincomycin/spectinomycin, penicillin, tetracyclines, tiamulin, trimethoprim/sulfamethoxazole, tylosin	[48]
Mexico	Ground beef	amoxicillin-clavulanic acid, carbenicillin, chloramphenicol, tetracyclines, trimethoprim-sulfamethoxazole	[49]
Thailand	Retail pork	Ampicillin, streptomycin, tetracyclines	[50]
United States	Retail meat	Amoxicillin-clavulanate, ampicillin, cefoxitin, ceftiofur, ceftriaxone, tetracyclines, gentamicin, streptomycin	[51]

**Table 3:** *Staphylococcus* spp. isolated from animal-derived foods and their resistance to antibiotics.

Species	Country	Sources	Antibiotic Resistance	References
*Staphylococcus aureus*	Brazil	Cow milk	ampicillin, cefoxitin, ceftiofur, clindamycin, erythromycin, oxacillin, penicillin, streptomycin, teicoplanin	[52]
	China	Raw cow milk	Clindamycin/norfloxacin, erythromycin, gentamicin, tetracyclines,	[53]
	Japan	Raw cow milk	Ampicillin, oxacillin, cefazolin, enrofloxacin, gentamicin, kanamycin	[54]
	South Africa	Raw meat	Erythromycin, oxacillin/cefoxitin, penicillin, tetracyclines	[41]
	Thailand	Fresh pork	Ampicillin, tetracyclines, vancomycin	[55]
	United States	Pork, beef, turkey, and chicken	Clindamycin, dalfopristin/quinupristin, erythromycin, gentamicin, levofloxacin, mupirocin, oxacillin, penicillin, tetracyclines	[56]
Methicillin-resistant *S. aureus* (MRSA)	Brazil	Cow milk	Ampicillin, erythromycin, oxacillin, penicillin, tetracyclines	[57]
	China	Bovine milk	Amoxicillin, ampicillin, cefoxitin, ceftiofur, cefuroxime, ciprofloxacin, clarithromycin, clindamycin, penicillin, sulfadiazine sodium	[58]
	Denmark	Retail food products (chicken, turkey, and pork)	Macrolides, penicillin, tetracyclines	[59]
	Egypt	Retail chicken	Amikacin, amoxicillin, ampicillin, chloramphenicol, ciprofloxacin, cloxacillin, erythromycin, gentamicin, netilmicin, penicillin, rifampicin, streptomycin, sulfamethoxazole-trimethoprim, tetracyclines, vancomycin	[60]
	Iran	Raw meat (beef, sheep, and goat)	Amoxicillin-clavulanic acid, ampicillin, azithromycin, ceftriaxone, clindamycin, cotrimoxazole, erythromycin, gatifloxacin, lincomycin, minocycline, oxacillin, penicillin G, tetracyclines	[61]
	United States	Pork, beef, turkey, and chicken	Cefoxitin, clindamycin, dalfopristin/quinupristin, erythromycin, gentamicin, levofloxacin, oxacillin, penicillin, tetracyclines	[56]
*S. aureus* and MRSA	China	Retail yak butter	amoxicillin/clavulanic acid, ampicillin, cefoperazone, cefoxitin, erythromycin, gentamicin, oxacillin, penicillin, sulfamethoxazole, tetracycliness, trimethoprim	[62]

**Table 4:** *Listeria* spp. isolated from animal-derived foods and their resistance to antibiotics.

Species	Country	Sources	Antibiotic Resistance	References
*Listeria* spp.	Iran	Raw milk and traditional dairy products	Amoxicillin/clavulanic acid, chloramphenicol, penicillin, tetracyclines	[63]
	Spain	Meat and dairy products	Ciprofloxacin, clindamycin, tetracyclines	[64]
*Listeria monocytogenes*	China	Pork, fish, sheep casing, chicken, and beef	Chloramphenicol, clindamycin, oxacillin, tetracyclines	[65]
	Indonesia	Chicken carcass	Ampicillin, erythromycin, penicillin	[66]
	Japan	Chicken meat	Cefoxitin, clindamycin, flomoxef, fosfomycin, linezolid, oxacillin	[67]
	Poland	Ready-to-eat food (heat-treated sausages and delicatessen), raw meat, raw sausages, and seafood (Fish and shrimp).	Ceftriaxone, ciprofloxacin, clindamycin, gatifloxacin, gentamycin, linezolid, oxacillin, tetracyclines	[68]
	Romania	Ready-to-eat food (sausages and ham), minced pork, and cheeses	Benzylpenicillin, ciprofloxacin, clindamycin, fosfomycin, fusidic acid, imipenem, oxacillin, rifampin, tetracyclines, trimethoprim-sulfamethoxazole	[69]
	Turkey	Chicken meat and beef	Ampicillin, ceftriaxone, clindamycin, fusidic acid, penicillin	[70]

**Table 5:** Essential oils (EOs) derived from aromatic plants and their antibacterial properties according to their minimum inhibitory concentration (MIC) values.

Family	Latin Name	Part Used	Extraction Method	Location	Main Constituents	Target Bacteria	Doses	MIC	References
Apiaceae	*Carum carvi* L.	Seeds	Hydro-distillation	Kelibia	γ-terpinene (31.03%), β-pinene (18.77%), *p*-cymene (17.16)	*E. coli*, *S. aureus*, *S.* Typhimurium, *Listeria monocytogenes*	-	0.469 mg/mL (*E. coli*, *L. monocytogenes*), 0.117 mg/mL (*S. aureus*), 0.234 mg/mL (*S.* Typhimurium)	[73]
	*Coriandrum sativum L.*	Seeds	Hydro-distillation	Kelibia	Linalool (76.41), γ-terpinene (5.35%), α-pinene (4.44%)	*E. coli*, *S. aureus*, *S.* Typhimurium, *L. monocytogenes*	-	0.938 mg/mL (*E. coli*, *L. monocytogenes*, *S.* Typhimurium), 0.234 mg/mL (*S. aureus*)	[73]
	*Foeniculum vulgare* Mill.	Seeds	Hydro-distillation	India	*trans*-anethole (50.4%), methyl chavicol (22.4%), limonene (11.4%)	*E. coli*, *S.* Typhimurium	0.0075–2.0% (*v*/*v*)	0.062% (*E. coli*), 0.031% (*S.* Typhimurium) (*v*/*v*)	[74]
Asteraceae	*Achillea millefolium* L.	Inflorescence, leaves, whole aerial parts	Hydro-distillation	India	Borneol (4.7–24.9%), sabinene (4.0–38.9%), germacrene D (1.1–46.6%)	*S. aureus*, *S. epidermidis*, *Klebsiella pneumoniae*	-	125–500 μg/mL	[75]
	*Helichrysum italicum* (Roth) G. Don	Inflo-rescence	Hydro-distillation	Central Europe	Neryl acetate (16.38%), nerol (15.73%), geraniol (6.32%)	*E. coli*, *S. aureus*, *Pseudomonas aeruginosa*	-	64 mg/mL (*E. coli*, *P. aeruginosa*), 1 mg/mL (*S. aureus*)	[76]
	*Helichrysum microphyllum* subsp. *tyrrhenicum*	-	Hydro-distillation	Iglesias	γ-curcumene (28.94%), linalool (14.21%), 5-eudesmen-11-ol (9.81%)	*E. coli*, *S. aureus*, *P. aeruginosa*	0.063–4 mg/mL	>4 mg/mL (*E. coli*, *P. aeruginosa*), 2 mg/mL (*S. aureus*)	[77]
Lamiaceae	*Origanum vulgare* L. spp.	*O. vulgare* L. ssp. *virens*	*n*-Hexane hydro-distillation	Southern Italy	Carvacrol (63.8%), γ-terpinene (7.4%), *p*-cymene (6.7%)	*E. coli*, *S. aureus*, *S.* Typhi	0.8–100 μg/mL	50 μg/mL (*E. coli*, *S. aureus*), 100 μg/mL (*S.* Typhi)	[78]
	*Rosmarinus officinalis* L.	Air-dried leaves	Steam distillation	Taizhou, Zhejiang	1,8-Cineole (26.54%), α-pinene (20.14%), camphor (12.88%), camphene (11.88%)	*S. aureus*, *S. epidermidis*, *Bacillus subtilis*	0.2–4% (*v*/*v*)	0.03–1.0% (*v*/*v*)	[79]
	*Thymus vulgaris*	Dried leaves	Hydro-distillation	North Yemen	Thymol (51.34%), *p*-cymene (18.35%), caryophyllene (4.26%), α-pinene (2.95%)	*E. coli*, *S. aureus*, *B. subtilis*, *Mycobacterium smegmatis*	0.01–30 mg/mL	0.075–1.1 mg/mL	[80]
	*Mentha pulegium* L.	Air-dried leaves	Steam distillation	Algerian	Pulegone (70.66%), neo-menthol (11.21%), menthone (2.63%)	*E. coli*, *S. aureus*, *B. subtilis*	0.3–20 μL/mL	1.25–10 μL/mL	[81]
	*Ocimum basilicum* L.	Leaves	Steam distillation	Ponta Grossa, Brazil	Linalool (55.2%), 1,8-cineole (8.8%), α-*trans*-bergamotene (7.0%), eugenol (3.2%)	*S. aureus*	2–1024 μg/mL	1024 μg/mL	[82]
	*Lavandula x intermedia* (lavandin) ‘Grosso’	Flowers, stems	Steam distillation	Lazio Region, Italy	Linalool (35.8%), 1,8-cineole (19.8%), α-pinene (8.7%)	*E. coli*, *B. cereus*	-	1.87% (*E. coli*), 0.94% (*B. cereus*) (*v*/*v*)	[83]
	*Chenopodium ambrosioides* L.	Leaves	Hydro-distillation	Crato, Brazil	α-terpinene (40.73%), *p*-cymene (21.81%), *trans*-ascaridol (12.48%)	*S. aureus*	0.5–1024 μg/mL	≥1024 μg/mL	[84]
Lauraceae	*Cinnamomum cassia* Blume	-	Hydro-distillation	China	Cinnamaldehyde (85.06%)	*E. coli*, *S. aureus*, *P. aeruginosa*, *Proteus vulgaris*, *Enterobacter aerogenes*, *Vibrio parahaemolyticus*, *V. cholerae*	-	75–600 μg/mL	[85]
	*Cinnamomum camphora* var. *linaloofera* Fujita	-	-	Guangzhou	Linalool (69.94%), camphor (10.90%), nerolidol (10.92%), safrole (8.24%)	*E. coli*	-	0.2 μL/mL	[86]
	*Litsea cubeba (Lour.) Pers.*	-	-	Caussols plateau, France	β-Citral (39.25%), α-citral (30.90%), limonene (8.28%), *trans*-verbenol (4.18%)	MRSA	0.125–4 mg/mL	0.5 mg/mL	[87]
Myrtaceae	*Eucalyptus globulus* L.	Aerial parts	Hydro-distillation	Takelsa	*p*-cymene (12.58–37.82%), α-pinene (10.41–13.39%), 1,8-cineole (7.71–13.23%), γ-terpinene (2.94–10.57%)	*S. aureus*, MRSA, *B. cereus*	-	1–4 mg/mL	[88]
	*Syzygium aromaticum*	Fresh leaves	Steam distillation	Nitra, Slovakia	Eugenol (82.4%), (*E*)-caryophyllene (14.0%), α-humulene (1.8%)	*S. aureus*	0.2–400 μL/mL	93.35 μL/mL	[89]
Poaceae	*Cymbopogon nardus*	Leaves	Cleavenge hydro-distillation	Ceara’, Brazil	Geraniol (33.88%), citronellal (27.55%), citronellol (14.40%), carvone (10.06%)	*S. aureus*, *E. coli*	0.125–8 mg/mL	0.5 mg/mL (*S. aureus*), >8 mg/mL (*E. coli*)	[90]
Rutaceae	*Citrus limon* L. Burm.	Peels	-	Sichuan Province	Limonene (48.48%), β-terpinene (17.08%), 4-carene (8.46%)	*S. mutans*	2.25–9 mg/mL	4.5 mg/mL	[91]
Zingiberaceae	*Alpinia pahangensis* Ridl.	Rhizomes	Hydro-distillation	Pahang, Peninsular Malaysia	γ-selinene (11.60%), β-pinene (10.87%), (*E*,*E*)-farnesyl acetate (8.65%), α-terpineol (6.38%)	*S. aureus*	0.039–5 mg/mL	<0.31 mg/mL	[92]

**Table 6:** Individual compounds of common EOs and their antibacterial properties according to their MIC values.

Item	Individual Compounds	Chemical Structures	Target Bacteria	Doses	MIC	References
Terpenes	β-Caryophyllene	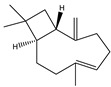	*E. coli*, *S. aureus*	0.1–4 mg/mL	>4 mg/mL	[93]
	Limonene	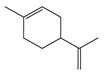	*E. coli*, *S. aureus*, *S.* Typhimurium, *B. cereus*	0.002–0.25 mg/mL	0.25 mg/mL (*E. coli*, *S. aureus*, *B. cereus*), 0.06 mg/mL (*S.* Typhimurium)	[94]
Terpenoids	Borneol	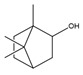	*E. coli*, *S.* Typhimurium, *S. aureus*, *B. cereus*	0.002–0.25 mg/mL	0.25 mg/mL (*E. coli*), 0.03 mg/mL (*S. aureus*), 0.12 mg/mL (*B. cereus*, *S.* Typhimurium)	[94]
	Camphor	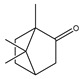	*E. coli*, *S.* Typhimurium, *S. aureus*, *B. cereus*	0.002–0.25 mg/mL	0.25 mg/mL (*E. coli*, *S.* Typhimurium, *B. cereus*), 0.015 mg/mL (*S. aureus*)	[94]
	Carvacrol	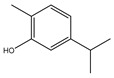	*E. coli*, MRSA, *S. mutans*, *Aggregatibacter actinomycetemcomitans*	-	0.4 mg/mL (*E. coli*, MRSA, *S. mutans*), 0.2 mg/mL (*A. actinomycetemcomitans*)	[95]
			*E. coli*, *Salmonella* spp., *Clostridium perfringens*	0.075–2 mg/mL	>0.6 mg/mL	[96]
			*S. aureus*	0.05–3.2 mg/mL	>0.4 mg/mL	[97]
	Citral	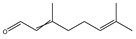	*E. coli*, *S.* Typhimurium, *S. aureus*, *B. cereus*	0.002–0.25 mg/mL	0.06 mg/mL (*E. coli*, *S. aureus*, *B. cereus*), 0.07 mg/mL (*S.* Typhimurium)	[94]
	Citronellal	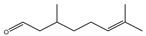	*E. coli*, *S. aureus*	-	0.3 mg/mL (*E. coli*), 0.4 mg/mL (*S. aureus*)	[98]
	Citronellol	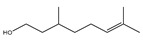	*E. coli*, *S. aureus*	-	0.005 mg/mL (*E. coli*), 0.375 mg/mL (*S. aureus*)	[98]
	Farnesol	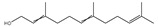	*Cutibacterium acnes*	0.004–0.576 μmol/mL	0.14 μmol/mL	[99]
	*trans*-Geraniol	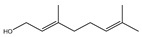	*E. coli*, *S. aureus*, *S.* Typhimurium, *B. cereus*	0.002–0.25 mg/mL	0.06 mg/mL (*E. coli*), 0.03 mg/mL (*S. aureus*, *S.* Typhimurium), 0.07 mg/mL (*B. cereus*)	[94]
	Linalool	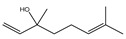	*E. coli*, *S. aureus*, *S.* Typhimurium, *B. cereus*	0.002–0.25 mg/mL	0.25 mg/mL	[94]
	Menthone	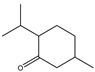	*K. pneumoniae*	-	224 mg/mL	[100]
	Nerolidol	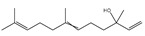	*S. aureus*, *K. pneumonia*, *P. aeruginosa*	-	2 mg/mL (*S. aureus*), 0.5 mg/mL (*K. pneumonia*, *P. aeruginosa*)	[101]
	Thymol	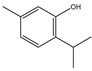	*E. coli*, MRSA, *A. actinomycetemcomitans*, *S. mutans*	-	0.2 mg/mL (*E. coli*, MRSA, *S. mutans*), 0.1 mg/mL (*A. actinomycetemcomitans*)	[95]
			*E. coli*, *C. perfringens*, *Salmonella* spp.	0.075–2 mg/mL	>1.2 μL/mL	[96]
			*S. aureus*	0.05–3.2 mg/mL	>0.8 mg/mL	[97]
Phenylpropanoids	Cinnamaldehyde	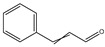	*E. coli*, *S. aureus*, *B. cereus*, *Yersinia enterocolitica*	-	5 mg/mL (*E. coli*), 1.875 mg/mL (*S. aureus*), 2 mg/mL (*B. cereus*), 5 mg/mL (*Yersinia enterocolitica*)	[102]
			*E. coli*, *C. perfringens*, *Salmonella* spp.	0.075–2 mg/mL	>0.6 μL/mL (*E. coli*, *Salmonella* spp.), >0.3 μL/mL (*C. perfringens*)	[96]
	Eugenol	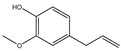	*E. coli*, *S. aureus*	0.1–4 mg/mL	0.4 mg/mL (*E. coli*), 1.3 mg/mL (*S. aureus*)	[93]
			*Campylobacter* spp.	-	0.5 mg/mL	[103]

**Table 7:** Summary of antibiotic resistance genes and corresponding resistance phenotype in *E. coli*, *Salmonella* spp., *Staphylococcus* spp., and *Listeria* spp.

Species	Resistance Genes and Types of Antibiotics or Antimicrobial Groups	References
*E. coli*	*bla*_TEM_, *bla*_OXA-1_: ampicillin, cefotaxime; *cat1*, *cat2*, *cmlA*: chloramphenicol; *sul1*, *sul2*, *folP*: sulfonamides; *tet*(A), *tet*(B): tetracyclines; *aphA1*, *aphA2*: kanamycin; *aadA1*: streptomycin; *aac(3)-IV*: gentamicin; *gyrA* (Ser83Leu, Asp87Asn), *gyrB* (Asp426Asn), *parC* (Ser80Ile), *parE* (Leu445His): quinolone; *pmrA* (Arg81His, Glu106Ala), *pmrB* (Gly206Arg, Tyr222His): colistin; *rpoB* (Ile572Phe): rifamycin; 23S: macrolides; 16S *rrsB*: gentamicin, spectinomycin, tetracyclines; 16S *rrsH*: spectinomycin	[31,35,38,133,134]
*Salmonella* spp.	*bla*_TEM-1_, *bla*_TEM-117_, *bla*_TEM-135_, *bla*_CTX-M-9_, *bla*_CTX-M55_, *bla*_CYM-2_: β-lactams; *gyrA* (Ser83Tyr, Asp87Asn), *gyrB* (Tyr420Cys), *parC* (Ser80Arg), *parE* (Ser458Pro): quinolone; *pmrB* (Val164Met, Arg92Pro): colistin; *sul1*, *sul2*, *sul3*: sulfonamides; *tet*(A), *tet*(B), *tet*(C), *tet*(G), *tet*(M), *tet*(R): tetracyclines; *dfrA1*, *dfrA12*, *dfrA14*: trimethoprim; *floR*, *cmlA1*: chloramphenicol; *aac(6′)-I*, *strA*, *strB*, *aadA (ant(3”)Ia*), *aac3-VIa*, *aph(3′)Ib*, *aphA (aph(3′)IIa)*, *aac3-IId*, *aadB (ant(2”)Ia), aac-IVa*, *aph(4)Ia*: aminoglycosides; *fosA2*: fosfomycin; *mph*(A), *ere*(A), *mef*(B): macrolides; *arr2*: rifampicin	[135,136,137,138]
*Staphylococcus* spp.	*blaZ*, *mecA*, *mecC*: β-lactams; *erm*(A), *erm*(C): erythromycin, clindamycin; *mphC*, *msrA*: erythromycin; *aacA-aphD*: gentamicin; *aadD*: tobramycin; *fusB*, *fusC*: fusidic acid; *tet*(K), *tet*(L), *tet*(M): tetracyclines; *fexA*: chloramphenicol; *fosB*: fosfomycin; *inuA*: lincomycin; *vanA*: vancomycin; *msr*(A), *mph*(C): macrolides, lincosamides, streptogramins	[60,139,140,141]
*Listeria* spp.	*bla*_TEM_, *bla*_CTX-M-9_: β-lactams; *tet*(A), *tet*(B), *tet*(C), *tet*(M), *tet*(O), *tet*(S): tetracyclines; *strA*, *aadA*, *aadB*, *ant6*: aminoglycosides; *dfrD*: trimethoprim; *sul1*, *sul2*: sulfonamides; *erm*(B): macrolides; *fos*(X), *vga*(D): lincosamides	[142,143,144]

**Table 8:** Nanoencapsulated EOs and individual compounds with anti-biofilm activity.

Essential Oils or Components	Emulsifier/Carrier System	Target Bacteria	Antibacterial Effects	References
Carvacrol	Polylactic acid nanoemulsions	*E. coli*, MRSA, *Acinetobacter baumannii*	Biofilm eradication	[180]
Cinnamon oil	Liposomes (average particle size: 144.3 nm)	MRSA	Biofilm eradication	[181]
Cinnamon oil, eucalyptus oil, orange oil	Mesoporous silica nanoparticles	*S. aureus*, *E. coli*	Inhibition of biofilm formation	[175]
Citral	Nanoemulsions (tween 80)	*L. monocytogenes*	Inhibition of biofilm formation	[182]
Eucalyptus oil	Silica nanoparticles	*E. coli*	Biofilm eradication	[164]
Eugenol	Nanoemulsions (tween 80, medium-chain triglyceride)	*P. aeruginosa*	Inhibition of biofilm formation	[45]
Lemongrass oil	Nanoemulsions (tween 80)	*Enterococcus faecalis*	Inhibition of biofilm formation	[183]
Limonene	Nanoemulsions (tween 80, propylene glycol)	MRSA	Reduction in biofilm persistence	[184]
Mandarin oil	Chitosan nanoparticles	*S. aureus*, *E. coli*	Inhibition of biofilm formation	[185]
Oregano oil	Biological silver nanoparticles	*S. aureus*	Decrease in cell density and exopolysaccharide matrix	[186]
Peppermint oil, cinnamaldehyde	Silica nanocapsules	*E. coli*, *S. aureus*, *P. aeruginosa*	Biofilm eradication	[187]
Tea tree oil	Nanostructured lipid carriers (average particle size: 166 nm)	*P. aeruginosa*	Decrease in adhesion and inhibition of biofilm formation	[188]
Thyme oil	Nanoarchaeosomes (made by soybean phosphatidylcholine, total polar archaeolipids and polysorbate 80), nanoliposomes (made by soybean phosphatidylcholine and polysorbate 80)	*S. aureus*	Biofilm eradication, inhibition of biofilm formation	[189]
Thyme oil, thymol	Chitosan nanoemulsions	*S. aureus*, *E. coli*	Inhibition of biofilm formation	[190]

## Data Availability

All of the data is contained within the article.
